# Reviewers

**DOI:** 10.1111/jdi.12923

**Published:** 2018-11-04

The publication of invaluable papers in the Journal of Diabetes Investigation depends on the prompt, careful review of submitted manuscripts. We would like to thank the Editorial Board members, the International Editorial Board members, the Assistant Editorial Board members and the following experts for reviewing manuscripts from September 1, 2017 to August 31, 2018.

Norio Abiru

AA Adane

Toru Aizawa

Hiroshi Akazawa

Mohamed Al‐Shabrawey

Sirajudeen S. Alavudeen

Shin Amemiya

Keizo Anzai

Hidenori Arai

Atsushi Araki

Shin‐ichi Araki

Hiroyoshi Ariga

Rabia Arshad

Noriko Asahara

Shunichiro Asahara

Yoshimasa Aso

Koichiro Azuma

Tetsuya Babazono

Yuqian Bao

Fabrizio Barbetti

C. Bellanne‐Chantelot

Bishwajit Bhowmik

H. Bihan

Aldo Bonaventura

Edward Boyko

Michela Brambatti

Rebecca J. Brown

Lu Cai

Xiaoling Cai

Nigel A Calcutt

Soner Cander

Juliana Chan

TF Chan

HC Chang

Jer‐Ming Chang

Sanjay Chatterjee

Chao Chen

Cheng‐Hsu Chen

Gang Chen

Harn‐Shen Chen

Huang‐Chi Chen

Judy Chen

Jui‐Hung Chen

Liming Chen

Wei Chen

Yi‐Chuan Chen

Zhuo Chen

MT Chiang

Ken Chiu

Dong Hyeok Cho

Eun‐Hee Cho

Jae Hyoung Cho

Kae Won Cho

Sung Hee Choi

Yong Jun Choi

Chih‐Hsun Chu

NF Chu

Yanhui Chu

Tsung‐Ju Chuang

Daisuke Chujo

Sookja Chung

Cinzia Ciccacci

Rosaly Correa‐de‐Araujo

La Monte Suzanne M. de

Takahisa Deguchi

Hu Ding

Jörg Dirmaier

G. Drews

Ken Ebihara

Nathan Efron

Masanori Emoto

Suneng Fu

Xiaobing Fu

Rumi Fujikawa

Junji Fujikura

Kei Fujimoto

Shimpei Fujimoto

Shiho Fujisaka

Tomomi Fujisawa

Yukihiro Fujita

Yoshio Fujitani

Kei fukami

Astunori Fukuhara

Michiaki Fukui

Nobuaki Funahashi

Shinya Furukawa

Joana Gaspar

Denise Tavares Giannini

Javier P. Gisbert

Christian S. Goebl

Ali Gol

Atsushi Goto

Honglei Guo

Pawel Gutaj

Kyoung Hwa Ha

Kazuyuki Hamaguchi

Tomoya Hamaguchi

Yoshiyuki Hamamoto

Hidetaka Hamasaki

Maryam Hami

Ko Hanai

Akinori Hara

Kazuo Hara

Mariko Harada‐Shiba

Naotake Hashimoto

Yoshitaka Hayashi

Yasuaki Hayashino

Tatsuhito Himeno

Tsutomu Hirano

Takumi Hirata

Takahisa Hirose

Yushi Hirota

Yukio Horikawa

Junichi Hoshino

Masayuki Hosoi

Tetsuya Hosooka

Kikuko Hotta

Chang‐Hsun Hsieh

Hui‐Min Hsieh

Bang‐Gee Hsu

Gang Hu

Chia‐Luen Huang

Qiaobing Huang

Elaine Hui

You‐Cheol Hwang

Chii‐Min Hwu

Kaori Ikeda

Hiroshi Ikegami

Takeshi Inagaki

Reiko Inagi

Toyoshi Inoguchi

Ikuo Inoue

Kazuo Inoue

Junichiro Irie

Nigel Irwin

Yasushi Ishigaki

Hisamitsu Ishihara

Yuichi Ishihara

Hiromi Iwahashi

Naoko Iwasaki

Masanori Iwase

Tetsuro Izumi

Tina Jafari

Hak Chul Jang

Edward Janus

Jae‐Han Jeon

Masahiro Kamata

Hideki Kamiya

Kyuzi Kamoi

Masahiro Kamouchi

Keizo Kanasaki

Akio Kanazawa

Ippei Kanazawa

Shizuka Kaneko

Hideaki Kaneto

Hideki Katagiri

Naoto Katakami

Koichi Kato

Noriko Kato

Vivian Kawai

Takahiko Kawamura

Tomoyuki Kawamura

Daiji Kawanami

Eiji Kawasaki

Arion Kennedy

Jothydev Kesavadev

Yoshiaki Kido

Bu Kyung Kim

Chong Hwa Kim

Chul‐Hee Kim

Eun Ky Kim

Jaetaek Kim

Nan Hee Kim

Sang Soo Kim

Sung‐Hoon Kim

yuji Kim

Tadahiro Kitamura

M Kizilgul

Kyung Soo Ko

Tetsuro Kobayashi

Motoyuki Kohjima

Shigeo Koido

Hitoshi Koito

Mitsuhisa Komatsu

Bokyung Koo

Daisuke Koya

Hidenori Koyama

Batya Kristal

Bon Jeong Ku

Akira Kubota

Naoto Kubota

Shinji Kume

Akio Kuroda

Takeshi Kurose

Akira Kurozumi

Hitoshi Kuwata

Soo‐Heon Kwak

Ting‐I Lee

Chien‐Nan Lee

I‐Te Lee

Jia‐Jung Lee

Kwan‐Woo Lee

Myung‐Shik Lee

Yau‐Jiunn Lee

Duo Li

Hong Li

Hung‐Yuan Li

Jun Li

Raymond Li

Xia Li

Xinjun Li

Yanbing Li

Jun Liang

Chia‐Huei Lin

Zhenqi Liu

Ming Lu

Peirong Lu

Jianhua Ma

Norikazu Maeda

Shiro Maeda

Hiroshi Maegawa

S Makino

Yamanouchi Masayuki

Kunio Masumoto

Hiroaki Masuzaki

Ikuro Matsuba

Masafumi Matsuda

Munehide Matsuhisa

Munetaka Matsuhisa

Michihiro Matsumoto

Takeshi Matsumura

Claire L. Meek

Otsuki Michio

Takashi Miida

Takashi Miki

Koki Mise

Hirofumi Misu

Tomoya Mita

Junnosuke Miura

Yoshihiro Miyake

Takeshi MIyatsuka

Hideaki Miyoshi

Tetsuya Mizoue

Hiroki Mizukami

Jong‐Seok Moon

Katsutaro Morino

Tomoaki Morioka

Chieko Murakami

Koji Murao

Takashi Murata

Toshinori Murata

Yoshio Nagai

Mototsugu Nagao

Shoichiro Nagasaka

Tomoko Nakagami

Atsushi Nakagawa

Takahiko Nakagawa

Akinobu Nakamura

Shuhei Nakanishi

Masamitsu Nakazato

Yoshihumi Narumi

Masami Nemoto

Rimei Nishimura

Wataru Nishimura

Akiko Noda

Mitsuhiko Noda

Takashi Nomiyama

Masatoshi Nomura

Shinsuke Noso

Makiko Ogata

Masahito Ogura

Ken Ohashi

Mitsuru Ohsugi

Yasuharu Ohta

Yosuke Okada

Shiki Okamoto

Hiroshi Onuma

Haruhiko Osawa

Tsuguhito Ota

Thomas Pannicke

Kyong Soo Park

Zheng‐Rong Peng

Milan Perovic

Francesco Perticone

John Pickup

Enrique Reyes‐Munoz

Sang Youl Rhee

Maricruz Rivera‐Hernandez

Yoshifumi Saisho

Kazuhiko Sakaguchi

Juro Sakai

Masaya Sakamoto

Hiroshi Sakaue

Hiromi Sanada

Takayoshi Sasako

Hiroaki Satoh

Jo Satoh

Shinobu Satoh

DA Schoenaker

Yusuke Seino

Hetal Shah

Viral Shah

Yunfeng Shen

Kenichi Shikata

Satoru Shikata

Akira Shimada

Seiya Shimoda

Iichiro Shimomura

Shigeki Shinba

Jun Shirakawa

Steven Shoelson

John H. Sloan

Hirohito Sone

Masakatsu Sone

Norbert Stefan

Jasminka Stefulj

Jian‐bin Su

SC Su

Takayoshi Suganami

H. Sugimori

Kazushi Sugimoto

Takashi Sugiyama

Sunghwan Suh

Kan Sun

Atushi Suzuki

Ryou Suzuki

Kiyoshi Suzuma

Daniel Swerdlow

Yuji Tajiri

Mitsuyoshi Takahara

Toshinari Takamura

Shin Takasawa

Tomozumi Takatani

Kyosuke Takeshita

Fumihiko Takeuchi

Yoshinori Takeuchi

Sidney Tam

Yoshikazu Tamori

Yoshifumi Tamura

Katsuya Tanabe

Daisuke Tanaka

Nagaaki Tanaka

Shoichiro Tanaka

Ichiro Tatsuno

Yasuo Terauchi

Sebastien Theriault

Chitoku Toda

Ryuji Toh

Yoshihara Tokita

Masao Toyoda

Kang‐Ting Tsai

PC Tsiotra

Kyoichiro Tsuchiya

Tetsuro Tsujimoto

Shin Tsunekawa

Kazuyo Tsushita

Yasuko Uchigata

Shinichiro Ueda

Kohjiro Ueki

Hiroyuki Umegaki

Toshitaka Umemura

Hiroyuki Unoki‐Kubota

Tatsuhiko Urakami

Takashi Uzu

Roxana Valdes‐Ramos

Jun Wada

Chaofan Wang

Chi Chiu Wang

Chih Yuan Wang

JS Wang

Elizabeth Witherden Witherden

Kyu Chang Won

Chung‐Ze Wu

Shouzhu Xu

Wang‐Hong Xu

Wen Xu

Hiroaki Yagyu

Kentaro Yamada

Satoru Yamada

Tamaki Yamada

Yosuke Yamada

Yuichiro Yamada

Kazuya Yamagata

Sho‐ichi Yamagishi

Ritsuko Yamamoto‐Honda

Takeshi Yamamoto

Yasuhiko Yamamoto

Shunsuke Yamane

Masayuki Yamanouchi

Li Yan

T‐Y Yang

Hiroshi YATSUYA

A. Ozgur Yeniel

Rose Yeung

Norihide Yokoi

Hirohide Yokokawa

Masashi Yoneda

Sho Yoneda

Hideto Yonekura

Shin Yonemitsu

Kun‐Ho Yoon

Tohru Yorifuji

Shigeo Yoshida

Narihito Yoshioka

Mehmet Aytac Yuksel

Jae‐Seung Yun

Yang Ze‐Min

Dongfeng Zhang

Ping Zhang

Xiaolong Zhao

Jian Zhou

He‐qun Zou

